# Coexistence of Emphysematous Pyelonephritis and Cystitis in a Patient with COVID-19 Complicated with Spontaneous Pneumomediastinum

**DOI:** 10.1155/2021/3115644

**Published:** 2021-10-05

**Authors:** Ibrahim Boukhannous, Anouar El Moudane, Mehdi Chennoufi, Mohamed Mokhtari, Abdelghani Ouraghi, Hamza Mimouni, Ali Barki

**Affiliations:** ^1^Department of Urology, Mohamed VI University Hospital Center, Mohamed I University, Oujda, Morocco; ^2^Department of Anesthesia and Intensive Care, Mohamed VI University Hospital Center, Mohamed I University, Oujda, Morocco

## Abstract

We report the first case of a 52-year-old nondiabetic male admitted for management of uremic syndrome associated with emphysematous pyelonephritis (EPN), renal and perinephric abscess, and emphysematous cystitis (EC) on a single functional kidney with a large abundance of spontaneous pneumomediastinum (SP) complicating a SARS-CoV-2 pneumonia. The patient has benefited from several dialysis sessions, intravenous antibiotics, and percutaneous drainage. His clinical course was complicated by acute respiratory distress syndrome, and unfortunately, he died nine days following admission.

## 1. Introduction

Since the first case of COVID-19, no EPN and EC associated with SARS-CoV-2 pneumonia was reported, with only a few spontaneous pneumomediastinums reported in the literature. Here, we report the first association of these rare entities.

EPN is a necrotizing infection with gas presence in the renal parenchyma, collecting system, or perinephric tissues [1]. Meanwhile, EC is defined by the presence of gas inside and around the bladder wall produced by bacterial or fungal fermentation [2].

On the other hand, spontaneous pneumomediastinum in the SARS-CoV-2 pneumonia is a rare complication, defined by the presence of air in the mediastinum apart from any invasive or noninvasive positive pressure ventilation. Its pathophysiology is still poorly understood.

## 2. Case Report

A 52-year-old patient with no previous history was admitted to the emergency room for the management of acute renal failure with uremic syndrome. The clinical examination found a conscious patient, hemodynamically and respiratory stable, hypertensive at 190/100 mmgh, and febrile at 39°C with generalized abdominal defense, with a preserved diuresis. The admission assessment showed a creatinine level at 169 mg/l, a urea level at 4.75 g/l, K+ at 5.2 mmol/L, Na at 132 mmol/L, Ca at 91 mg/L, CRP at 240 mg/L, a white blood cell count at 12190 per microliter, hypochromic microcytic anemia at 8.5 g/dL, leukocyturia at 11860000 per microliter, and hematuria at 315000 per microliter.

**Figure 1:**
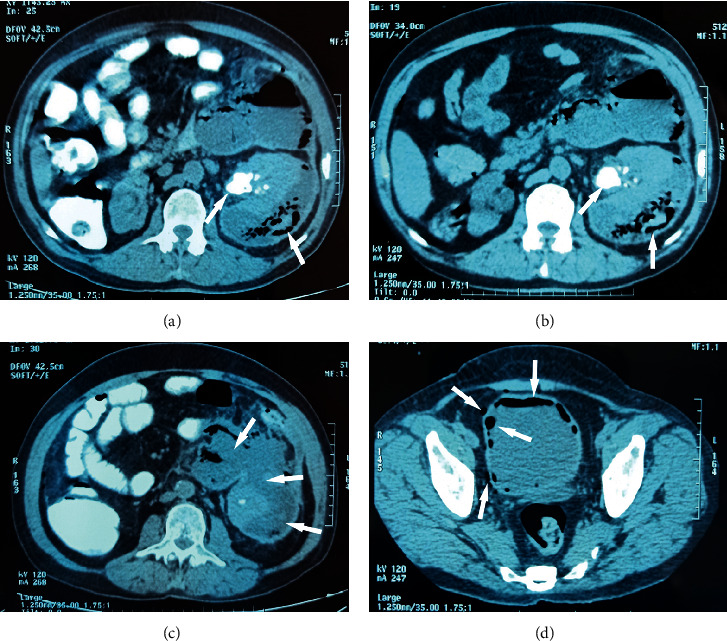
Abdominal CT scan showing (a, b) obstructive emphysematous pyelonephritis on a single functional kidney, (c) renal abscess communicating with intraperitoneal collections measuring approximately 208 × 113 mm with no passage of intraperitoneal contrast product at high digestive opacification, and (d) emphysematous cystitis.

An abdominal CT scan was carried out as part of the aetiological assessment objectified right kidney atrophy, and a left kidney sits with calyceal dilation upstream of a pyelic lithiasis of 29 × 21 mm density 500 HU with bubbles of intracaliceal air complicated by a collection under the left renal capsule, well limited, hypodense containing air bubbles, with enhanced thin wall after injection of contrast product, communicating with intraperitoneal collections at the level of the left iliac fossa, the left parietocolic groove, and the peripancreatic and periduodenal hypochondrium measuring approximately 208 × 113 mm with air bubbles at the level of the bladder. The high digestive opacification does not show any passage of the contrast product intraperitoneally (Figures 1(a)–1(d)).

The patient received 4 dialysis sessions, probabilistic antibiotic therapy based on ceftriaxone for 48 hours with adaptation by imipenem after ECBU result returning in favor of multidrug-resistant *Pseudomonas aeruginosa*. Furthermore, percutaneous drainage of the collection with a left double J stent placement with bladder irrigation was performed. The course was marked by the disappearance of the fever on the 4th day after admission and an improvement in the infectious assessment and renal function.

**Figure 2:**
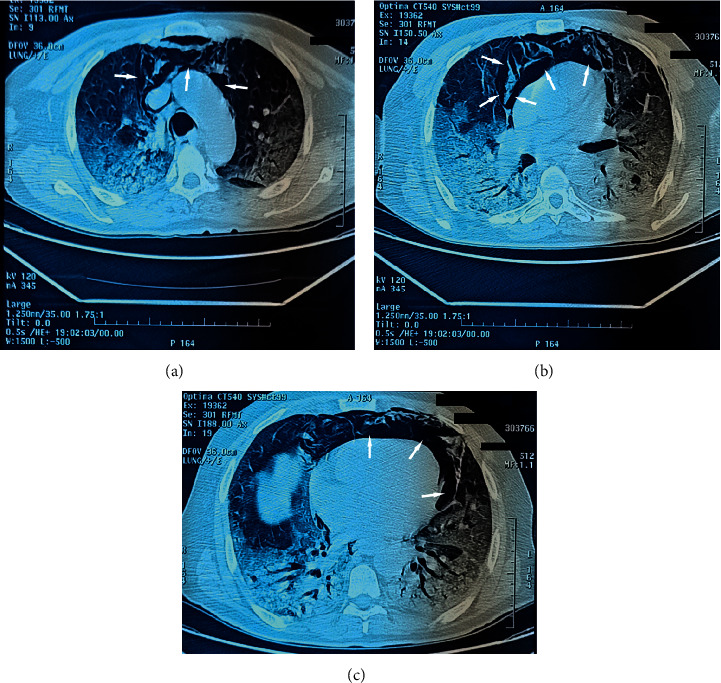
(a, b, c) Chest CT scan showing ground-glass opacities with crazy paving of mixed topography, bilateral to lower predominance with a degree of critical involvement >75% classified CORADS 5 and a large abundance of spontaneous pneumomediastinum.

However, on the 7th day, the patient presented with chest pain and respiratory distress made up of polypnea with an 84% desaturation requiring an increase in O2 intakes to 15 l/min with the mask at high concentration. After stabilization of the patient, a thoracic CT angiography was performed objectifying ground-glass opacities with crazy paving of mixed topography, bilateral to lower predominance with a degree of critical involvement >75% classified CORADS 5 and a large abundance of pneumomediastinum with no sign of pulmonary embolism (Figures 2(a)–2(c)). A PCR test is made positive. The patient was placed on Claforan 1 g/8 h, dexamethasone 6 mg/d, Rovamycin 3 MI/8 h, Lovenox 4000 IU/d, paracetamol 1 g/8 h, and omeprazole 40 mg/d.

Faced with no respiratory improvement, the patient was transferred to intensive care. The blood gas analysis found a hypoxic patient, pH at 7.34, PaO2 at 52 mmHg, PaCO2 at 32 mmHg, and bicarbonate at 30 mmol/l, initially put on Optiflow with FiO2 at 100%. Saturation improved to 92%.

Six hours after his admission to intensive care, a worsening of the polypnea with signs of respiratory struggles and thoracoabdominal swaying set in with 82% desaturation under Optiflow, hence the indication of protective ventilation intubation with tidal volume at 10 PEP, FR at 20 cycles/minute, and FiO2 at 70% with sedation by Diprivan 120 mg/h, Sufenta 15 gamma/h, and Atacurium 50 mg/h.

The course was marked by the cardiorespiratory arrest on D2 of hospitalization in intensive care following refractory hypoxia.

## 3. Discussion

The mortality rate of EPN or EC is between 8.7 and 21%. It is a life-threatening infection on its own [1, 3]. Several risk factors were associated with a higher mortality rate, such as shock condition, urgent hemodialysis, severe hypoalbuminemia, inappropriate antibiotic therapy, and polymicrobial infections [4].

Usually, it affects diabetic patients, which is not the case with this patient. Moreover, the high urinary sugar level provides a favorable environment for urinary-onset sepsis development, especially EPN, EC, renal, and perirenal abscess, which is already very rare [5]. Our patient is the first to have grouped this rare combination with SARS-CoV-2 pneumonia complicated by a large abundance of SP.

Based on the CT scan data, there are two classifications of EPN in the literature, Huang et al. and Wan et al.'s classifications [6].

Huang et al.'s classification proposed 4 classes:

- Class 1: emphysematous pyelitis
- Class 2: gas in the renal parenchyma without extension to the extrarenal space
- Class 3A: extension of the gas or abscess to the perinephric space
- Class 3B: extension of the gas or abscess to the pararenal space
- Class 4: bilateral EPN or solitary kidney with EPN

Furthermore, Wan et al.'s classification [7] proposed 2 types:

- Type I EPN: renal parenchymal with an absence of fluid content or the presence of streaky/mottled gas
- Type II EPN: the presence of renal or perirenal fluid accompanied by a bubbly gas pattern or the presence of gas in the collecting system

Our patient was classified as class 4 and type II EPN of Huang et al. and Wan et al.'s classifications, respectively.

For the initial management of type I EPN, emergent nephrectomy is recommended. However, percutaneous drainage is indicated for the treatment of type II EPN [8].

On the other hand, pneumomediastinum can be primary or spontaneous if the cause is idiopathic or secondary if it is caused by a traumatic or iatrogenic etiology [9]. Dyspnea and chest pain are the most common symptoms [10]. Predisposing factors such as drug abuse, asthma, and chronic obstructive pulmonary disease are proved, with tobacco being the most important [9].

The physiopathology of SP is not fully proven. The SARS-CoV-2 virus infects type II pneumocytes causing alveolar damage, generating interstitial emphysema that could progress to the mediastinal space and subcutaneous emphysema [11] compounded by the increasing pressure difference between the alveolar space and the interstitium [12].

To accelerate the resorption of pneumomediastinum, oxygen therapy, which is not systematic, would cause the absorption of free air by increasing the nitrogen concentrations [13].

The diffuse COVID-19 complicated by a large abundance of SP was associated with a severe clinical course characterized by sudden acute respiratory distress syndrome that required aggressive management and early intubation. Furthermore, the presence of pneumomediastinum required some changes in the management of mechanical ventilation to minimize volutrauma and prevent its expansion. Overventilation should be avoided, measures should be taken to limit hyperinflation, and low positive end-expiratory pressures should be used [14].

The diagnosis of pneumomediastinum in diffuse COVID-19 is associated with a grim prognosis [15].

## 4. Conclusions

This combination of EPN and EC in a patient with diffuse COVID-19 complicated by a large abundance of SP is the first case of its kind. Its management is multidisciplinary. The prognosis of each pathology is already bleak. Their combination is associated with a worse prognosis and fatal outcomes.
